# Triple-enrichment interface enabled by surfactant-modified carbon nanotube for signal-off electrochemiluminescence detection of antitumor drug mitoxantrone

**DOI:** 10.3389/fchem.2026.1897134

**Published:** 2026-07-23

**Authors:** Wuning Zhong, Jiahui Lin, Xianwei Mo, Fei Yan, Ke He

**Affiliations:** 1 Guangxi Medical University Cancer Hospital, Nanning, China; 2 Department of Chemistry, School of Chemistry and Chemical Engineering, Zhejiang Sci-Tech University, Hangzhou, China

**Keywords:** antitumor drug, electrochemiluminescence sensor, mitoxantrone, signal-off, surfactant-modified carbon nanotube

## Abstract

Accurate monitoring of antitumor drug is essential for therapeutic management and safety evaluation. Herein, an electrochemiluminescence (ECL) sensor was developed for sensitive detection of antitumor drug mitoxantrone (MIT) based on surfactant-modified carbon nanotubes. Cetyltrimethylammonium bromide (CTAB)-modified carboxylated multi-walled carbon nanotube (cMWCNTs) composite (CTAB@cMWCNTs) was prepared through a simple ultrasonic-assisted assembly method and immobilized onto indium tin oxide (ITO) electrode by drop-casting. By forming a stable interfacial modification layer, enhanced ECL response was likely attributed to the inferred interfacial accumulation of luminol (emitter) and dissolved oxygen (DO, co-reactant) on the CTAB@cMWCNTs-modified ITO electrode, as suggested by cyclic voltammetry (CV) and ECL results. MIT could consume key reactive oxygen species (ROS), decreasing ECL signal and leading to signal-off ECL sensing of MIT. Enrichment of CTAB@cMWCNTs towards MIT improved detection sensitivity. The proposed sensor exhibited wide linear response toward MIT from 100 pM to 100 μM, with low limit of detection (LOD, 4.8 pM). The sensor had good selectivity and reproducibility as well as high stability. Detection of MIT in human serum was also investigated. This work provides a facile and sensitive ECL strategy for monitoring antitumor drugs and highlights the potential of surfactant-modified carbon nanomaterials in bioanalytical applications.

## 1 Introduction

Accurate monitoring of drug concentrations is essential for therapeutic management and safety evaluation (Deng et al., 2023; Lv et al., 2022; Wei et al., 2022; Zhou et al., 2022). Mitoxantrone (MIT), an anthracenedione antineoplastic agent, is widely used for breast cancer, leukemia treatment, lymphoma, prostate cancer, and multiple sclerosis (Faulds et al., 1991). Its therapeutic activity is mainly associated with DNA intercalation and inhibition of topoisomerase II (Minko et al., 2024). However, MIT may cause dose-dependent adverse effects, especially cardiotoxicity and myelosuppression, which makes its accurate determination highly important in pharmaceutical quality control, therapeutic drug monitoring, and clinical safety assessment (Scott and Figgitt, 2004). Methods including high-performance liquid chromatography-mass spectrometry (HPLC) (An and Morris, 2010), capillary electrophoresis (CE) (Kika et al., 2009), and spectrophotometry (Zhong et al., 2023) have been used for MIT analysis. Although these methods provide satisfactory accuracy, they often require expensive instrumentation, time-consuming sample pretreatment, or skilled operators. Therefore, developing sensitive method for MIT detection is significant.

Electrochemiluminescence (ECL), which combines electrochemical excitation with luminescent signal, has attracted considerable attention in pharmaceutical analysis because of low background, wide detection range, and good controllability (Li et al., 2026; Lin et al., 2026a; Wu et al., 2025). Among various ECL emitters, luminol is widely used emitter because of low cost and well-established reaction pathway involving reactive oxygen species (ROS) (Cui et al., 2026; Liu et al., 2025; Song et al., 2024). Luminol-based ECL intensity can be effectively modulated by analytes that participate in electron-transfer reactions or interact with reactive intermediates, providing a feasible route for sensitive signal transduction (Zhang et al., 2025). For instance, MIT can scavenge ROS involved in the ECL process, a signal-off ECL sensing strategy can be developed for MIT detection. For the luminol–DO system, the efficient reactions between luminol and ROS mainly occur in the vicinity of the electrode/solution interface. Thus, the local concentrations of the ECL emitters and co-reactant are critical to the ECL intensity (Xia et al., 2022; Zhong et al., 2024). Improving the interfacial concentrations of analytes and promoting electron transfer at the electrode surface are also effective approaches to enhancing sensitivity for ECL detection (Ma et al., 2024; Shi et al., 2025; Yu et al., 2024). Modulating the interfacial microenvironment to simultaneously enrich emitter, co-reactant, and analyte together with accelerated electron transport remains highly desirable for improving ECL analytical performance (Fan et al., 2024; Huang et al., 2023; Luo et al., 2022).

Surfactants are a class of amphiphilic molecules that possess both hydrophilic and hydrophobic groups, and they exhibit unique interfacial adsorption properties. Cetyltrimethylammonium bromide (CTAB) is a typical cationic surfactant (Ghosh et al., 2020; Guerrero-Hernández et al., 2022). Its hydrophobic microdomains can effectively enrich nonpolar or weakly polar molecules, making it suitable for the accumulation of hydrophobic small molecules. In addition, it has been proven that O_2_ can be locally solubilized in the hydrophobic environment of CTAB (Hossain et al., 2021; Naccache and Shah, 1978). Luminol molecules, as ECL emitter, can also be enriched by CTAB (Lin et al., 2026b). Thus, a CTAB-modified interface is expected to realize a triple-enrichment effect toward the target analyte, the ECL emitter and the co-reactant. Carboxylated multi-walled carbon nanotubes (cMWCNTs) possess excellent electrical conductivity, large surface area, and abundant groups (e.g., oxygen-containing groups), allowing them to act as conductive nanowires to enhance interfacial electron transfer (Deline et al., 2020). Furthermore, CTAB can be assembled with cMWCNTs through electrostatic and hydrophobic interactions (Jose et al., 2012). The resulting composite integrates the enrichment capability of CTAB with the electron-transfer properties of cMWCNTs, making it promising for fabricating ECL sensing platforms with high-performance.

In this work, CTAB-modified cMWCNT composite was prepared for constructing ECL sensor to detect MIT. The enhanced ECL response was likely due to the interfacial accumulation of luminol, and dissolved oxygen on the CTAB@cMWCNTs-modified ITO electrode, which was inferred from the CV and ECL data. For MIT detection, CTAB@cMWCNTs interface further enriched MIT near the electrode surface. MIT consumed key ROS in ECL process, resulting in signal decrease. This signal-off mechanism was employed for sensitive detection of MIT.

## 2 Materials and methods

Chemicals and materials, measurements and instruments are provided in the Supplementary Material S1.

### 2.1 Preparation of CTAB@cMWCNTs

The CTAB@cMWCNTs composite was prepared using an ultrasonic-assisted assembly method. Briefly, cMWCNTs (10 mg) was dispersed in ultrapure water (500 mL) and ultrasonicated for 4 h. Then, CTAB (10 mg) was added followed with 30 min ultrasonication. After sonication, the solution was centrifuged at 6,000 rpm for 15 min. The precipitate was washed and redispersed in ultrapure water by ultrasonication. Finally, a CTAB@cMWCNTs dispersion (0.02 mg mL^-1^) was obtained.

### 2.2 Fabrication of CTAB@cMWCNTs/ITO electrodes

ITO glass (2.5 × 5 cm) was immersed in NaOH (1 M) overnight followed with clean in acetone, ethanol, and ultrapure water for 10 min each. 50 μL of CTAB@cMWCNTs dispersion was drop-cast onto clean ITO electrode (0.5 × 5 cm) and dried 30 min under infrared lamp. The resulting electrode was denoted as CTAB@cMWCNTs/ITO. For comparison, cMWCNTs/ITO and CTAB/ITO electrodes were prepared using the same procedure by replacing the drop-casting solution with a cMWCNTs dispersion and a CTAB solution, respectively.

### 2.3 ECL detection of MIT

CTAB@cMWCNTs/ITO electrode was used for MIT detection. Phosphate-buffered saline (PBS, 0.01 M, pH 7.4) containing 100 μM luminol was used as the electrolyte solution. ECL signals were generated and recorded by cyclic voltammetry (CV) scan (−1.0 to +0.8 V vs. Ag/AgCl). MIT concentration in human serum samples was determined using standard addition method (approved by the Ethics Committee of Guangxi Medical University Cancer Hospital, Approval Number: KY2024783). Specifically, the serum samples were used without any special pre-treatment. After the addition of MIT standard solutions at different concentrations, the spiked serum samples were diluted 50 times with electrolyte solution, and the MIT content was subsequently determined by ECL.

## 3 Results and discussion

### 3.1 Strategy for sensor fabrication and MIT detection

**FIGURE 1:**
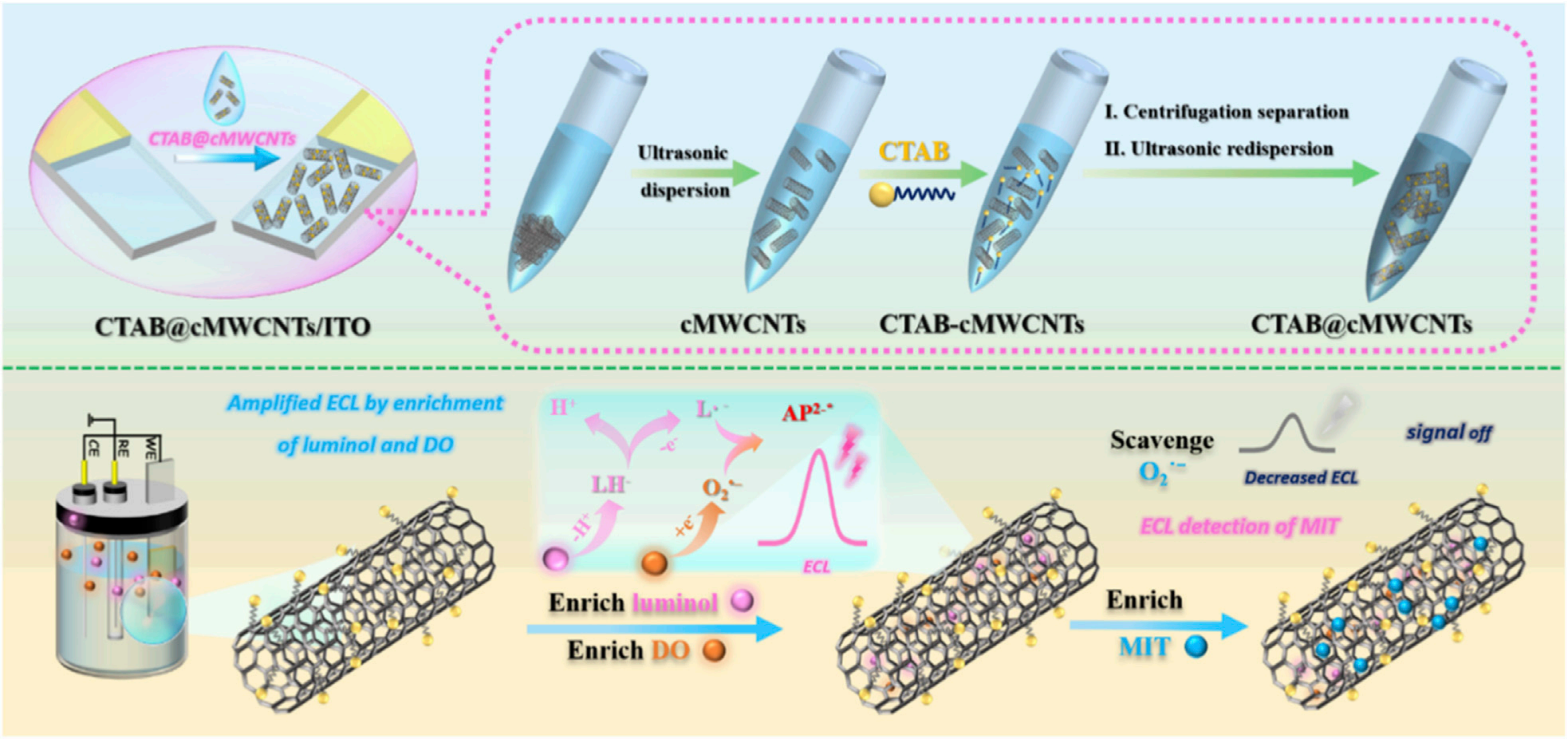
**(A)** Schematic illustration for preparation of CTAB@cMWCNTs composite and CTAB@cMWCNTs/ITO electrode. **(B)** Schematic representation of the CTAB@cMWCNTs/ITO electrode for ECL detection of MIT through the interfacial enrichment of luminol, DO, and MIT.

Figure 1A schematically illustrated the ultrasound-assisted preparation of the CTAB@cMWCNTs composite. Under ultrasonic treatment, cMWCNTs were hybridized with CTAB to form CTAB@cMWCNTs. Owing to oxygen-containing groups on cMWCNTs (e.g., -COOH and -OH), the nanotubes possessed a certain degree of negative surface charge. The cationic surfactant CTAB can therefore adsorb on cMWCNTs through electrostatic interactions. In addition, the hydrophobic alkyl chains of CTAB can also interact with the graphitic surface of cMWCNTs through hydrophobic interactions and *van der* Waals forces, leading to stable CTAB@cMWCNTs composites. After centrifugation, washing, and redispersion, unbound free CTAB was removed from the system, yielding a stable CTAB@cMWCNTs dispersion.

Figure 1B showed the preparation of CTAB@cMWCNTs/ITO electrode and the proposed mechanism for ECL detection of MIT. The CTAB@cMWCNTs/ITO modified electrode was prepared by simple drop-casting method, without requiring complicated modification procedures. The interfacial microenvironment constructed by CTAB might facilitate enrichment of luminol and DO at electrode surface, increasing their interfacial concentrations. Meanwhile, cMWCNTs might facilitate interfacial electron transfer. These effects resulted in a synergistic enhancement of ECL emission of luminol-DO. Upon introduction of the target analyte MIT, the CTAB@cMWCNTs composite promoted the accumulation of MIT at the electrode interface. MIT then consumes key reactive oxygen species involved in the ECL process, resulting in ECL decrease and enabling a signal-off ECL sensing mode.

### 3.2 Characterization of cMWCNTs and CTAB@cMWCNTs

**FIGURE 2:**
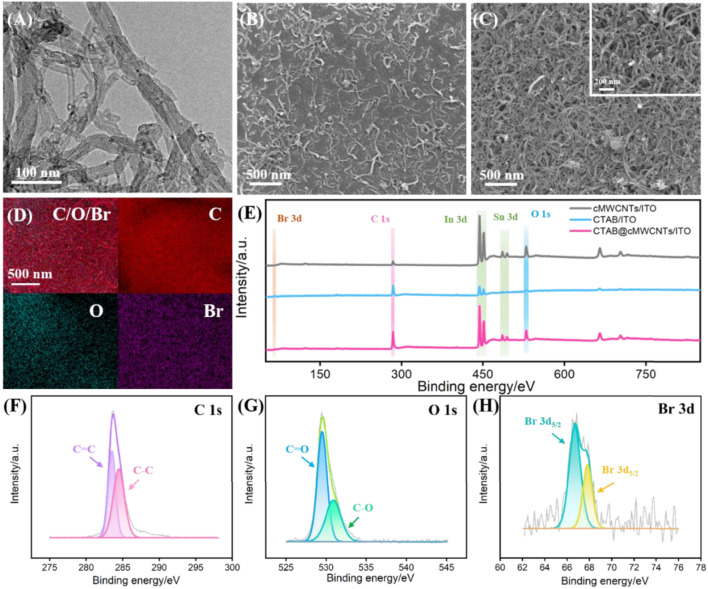
**(A)** TEM image of cMWCNTs. SEM images of **(B)** cMWCNTs and **(C)** CTAB@cMWCNTs. The inset was the magnified image **(D)** EDS elemental mapping images of CTAB@cMWCNTs. **(E)** XPS survey spectra of different electrodes. High-resolution XPS spectra of CTAB@cMWCNTs for **(F)** C 1 s, **(G)** O 1s, and **(H)** Br 3 days.

The morphologies of cMWCNTs and CTAB@cMWCNTs were characterized, as shown in Figures 2A–C. The transmission electron microscopy (TEM) image of cMWCNTs (Figure 2A) displayed typical multi-walled tubular structure with relatively uniform tube diameters and clearly defined tube walls. Due to strong *van der* Waals interactions, cMWCNTs were entangled and aggregated, forming a nanonetwork. Figures 2B,C showed scanning electron microscopy (SEM) images of cMWCNTs or CTAB@cMWCNTs modified electrodes, respectively. The cMWCNTs exhibited obvious entanglement and aggregation on the electrode surface. In contrast, CTAB@cMWCNTs showed remarkably improved dispersion on the electrode surface (Figure 2C), indicating that the introduction of CTAB effectively weakened nanotube aggregation, enhances dispersion, and improved the distribution of the composite on the electrode surface. As observed from high-magnification inset (Inset in Figure 2C), individual CTAB@cMWCNTs were more clearly distinguishable and formed a loose, homogeneous network structure.

To verify successful construction of CTAB@cMWCNTs, energy-dispersive X-ray spectroscopy (EDS) elemental mapping images were shown in Figure 2D. Br signal originating from CTAB showed a highly overlapped spatial distribution with C signal, confirming successful attachment of CTAB on cMWCNT. X-ray photoelectron spectroscopy (XPS) survey spectra of three electrodes, cMWCNTs/ITO, CTAB/ITO, and CTAB@cMWCNTs/ITO electrodes, and high-resolution spectra were shown in Figures 2E–H. C 1s peak was observed at approximately 284 eV, mainly originating from the carbon skeleton of cMWCNTs and the carbon chain structure of CTAB. As shown in Figure 2F, peaks at 284.4 and 285.1 eV were assigned to C=C and C–C bonds, respectively (Zhu et al., 2025). In addition, two intense peaks were observed at around 450 eV for all three electrodes were corresponded to In 3d5/2 and In 3d3/2 characteristic peaks of indium in the ITO substrate. Meanwhile, the peaks in the range of 486–495 eV were Sn 3d5/2 and Sn 3d3/2 orbitals of tin, originating from ITO electrode surface. As shown in Figure 2G, peaks at 530.5 and 532.2 eV were C=O and C–O bonds (Gu et al., 2026; Xu et al., 2023) on the cMWCNT surface, respectively. Furthermore, Br 3d5/2 and Br 3d3/2 characteristic peaks were observed at approximately 67 eV (Figure 2H). These results confirmed successful preparation of CTAB@cMWCNTs composite.

### 3.3 Enrichment of CTAB@cMWCNTs toward luminol and dissolved oxygen

**FIGURE 3:**
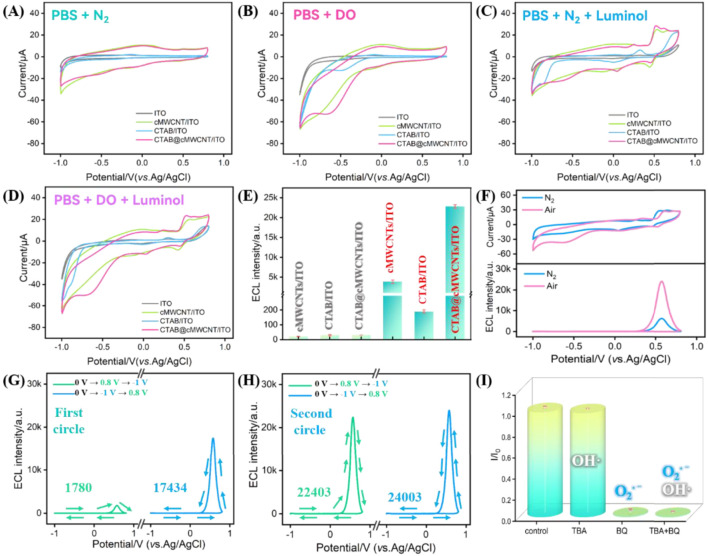
CV curves of different electrodes recorded in 0.01 M PBS (pH 7.4) under different conditions including **(A)** N_2_-saturated PBS, **(B)** non-deaerated PBS, **(C)** N_2_-saturated PBS with luminol (100 μM), and **(D)** non-deaerated PBS with luminol (100 μM). **(E)** ECL responses of cMWCNTs/ITO, CTAB/ITO, and CTAB@cMWCNTs/ITO electrodes in PBS immersion in PBS or luminol solution. **(F)** CV curves and ECL intensities of the CTAB@cMWCNTs/ITO electrode under different gas atmospheres. Relationship between ECL intensity and applied potential under different potential scan directions during **(G)** the first cycle and **(H)** the second cycle. **(I)** ECL responses of the luminol-containing 0.01 M PBS after adding radical scavengers. *I* and *I*_0_ represent ECL intensities without or with radical scavengers, respectively.

To investigate the roles of CTAB and CTAB@cMWCNTs, CV measurements were performed in 0.01 M PBS (pH 7.4). The effects of luminol and DO on the electrochemical behavior of different electrodes were systematically examined (Figures 3A–D). In N_2_-saturated PBS, no obvious reduction peak was observed in the cathodic potential region for ITO, cMWCNTs/ITO, CTAB/ITO, and CTAB@cMWCNTs/ITO electrodes (Figure 3A). In contrast, in non-deaerated PBS, all four electrodes exhibited distinct cathodic reduction peaks (Figure 3B), and the cathodic peak current at bare ITO electrode was much lower than those obtained at other electrodes. These results indicated that CTAB@cMWCNTs modified electrode exhibited an enhanced electrochemical response toward DO reduction. Notably, CTAB-containing modified electrodes showed a pronounced reduction peak at approximately −0.6 V, suggesting that the introduction of CTAB favors the local enrichment of DO near the electrode/solution interface, thereby enhancing the oxygen reduction response.

To further evaluate the interaction between the prepared electrodes and the emitter luminol, CV measurements were conducted after adding luminol to N_2_-saturated PBS. As shown in Figure 3C, all electrodes displayed obvious oxidation peaks in the anodic potential region. The oxidation peak of luminol at bare ITO electrode near +0.8 V was much weaker than those at the modified electrodes, whereas CTAB/ITO electrode exhibited a remarkedly enhanced oxidation signal compared with bare ITO. This result suggested that CTAB facilitated the local accumulation of luminol at electrode interface, improving its oxidation response. For cMWCNTs-modified electrodes, multiple oxidation peaks were observed, which may be associated with the multistep electrochemical oxidation of luminol on cMWCNTs surface. Finally, luminol was directly added to non-deaerated PBS for CV measurements (Figure 3D). These CV results demonstrate that CTAB@cMWCNTs/ITO integrated the interfacial enrichment capability of CTAB with the favorable properties of cMWCNTs, leading to a stronger DO reduction response during the cathodic scan and an enhanced luminol oxidation response during the anodic scan.

To further verify the enhancement effect of CTAB@cMWCNTs electrode on ECL of luminol-DO, cMWCNTs/ITO, CTAB/ITO, and CTAB@cMWCNTs/ITO electrodes were first immersed in PBS containing luminol and then transferred to PBS for ECL measurements. As a control, another set of electrodes was immersed in blank PBS and measured under identical conditions. As shown in Figure 3E, three electrodes immersed in blank PBS showed negligible ECL responses, whereas all electrodes pretreated with luminol-containing PBS exhibited low ECL signals. However, ECL intensity of CTAB/ITO electrode was only 193 a. u., probably because CTAB directly modified on the ITO surface has relatively weak binding stability and may partially detach during operation. In contrast, the CTAB@cMWCNTs/ITO composite electrode produced the strongest ECL signal. These CV and ECL results indicated that the CTAB@cMWCNTs composite interface can simultaneously enrich luminol and DO at the electrode surface and promote interfacial electron transfer, thereby significantly enhancing ECL response.

### 3.4 ECL mechanism of luminol-DO

Electrochemical behavior and ECL intensity of the CTAB@cMWCNTs/ITO electrode were investigated in luminol-containing PBS under different gas atmospheres. As shown in Figure 3F, under N_2_ atmosphere, the cathodic reduction current at −1.0 V was significantly lower than that recorded under air atmosphere, and the corresponding ECL signal was also much weaker. This phenomenon can be attributed to the cathodic reduction of O_2_, which generated abundant ROS and consequently produces a strong ECL emission (Fan et al., 2025; Fang et al., 2026; Zhou et al., 2024). An increased DO content in the system was favorable for generating active species at electrode interface, thereby enhancing ECL signal (Zhang et al., 2026). These results demonstrated that DO served as co-reactant in this system.

The influence of potential scan sequence on ECL signal was examined. As shown in Figure 3G, when the potential was scanned first in the anodic direction and then in the cathodic direction (0 V → +0.8 V → −1.0 V), ECL intensity on CTAB@cMWCNTs/ITO was small. In contrast, when the scan direction was changed (0 V → −1.0 V → +0.8 V), a much stronger signal was obtained. This behavior is closely related to the generation sequence and lifetime difference of the key reactive intermediates (Pei et al., 2025). During anodic process, luminol was oxidized to generate luminol anion radical (L^•−^), whose lifetime was generally shorter than that of ROS produced from DO reduction during the cathodic process. Thus, when cathodic scanning was performed first, a certain amount of ROS can be pre-generated and accumulated at the electrode interface, which subsequently reacted with newly generated L^•−^ during the anodic process, resulting in a much stronger ECL signal. Conversely, when anodic scanning was performed first, the initially generated L^•−^ cannot efficiently react with ROS produced later during the cathodic process, leading to a weak ECL response. To verify this phenomenon, two consecutive potential cycles were performed for both scan modes. ECL signals in the second cycle for the two scan modes were higher than those in the first cycle (Figure 3H). This further confirmed that the lifetime difference of the key radical intermediates was important in regulating the ECL response. The potential scan sequence significantly affected the ECL signal by modulating the generation order and interfacial accumulation of ROS and luminol-derived radical intermediates. Preferential cathodic-to-anodic scanning was more favorable for the pre-enrichment of relatively long-lived ROS at electrode surface, enabling their efficient reaction with subsequently generated L^•−^ and thus producing strong ECL signal.

To identify ROS generated during ECL process, scavenger (tert-butanol, TBA) for superoxide radicals (O_2_^•−^) and scavenger (benzoquinone, BQ) for hydroxyl radicals (•OH) were introduced (Huang et al., 2026; Li et al., 2022; Wu et al., 2026a; Wu et al., 2026b; Yan et al., 2026). Their effects on the ECL signal were shown in Figure 3I. The ECL intensity decreased significantly with BQ, whereas TBA caused no obvious change. When BQ and TBA were added simultaneously, the decrease in ECL intensity was nearly identical to that observed with BQ alone. These results clearly indicated that O_2_^•−^ was the dominant ROS in ECL reaction of luminol–DO.

Thus, ECL mechanism of luminol-DO system at CTAB@cMWCNTs/ITO electrode can be proposed as follows (Equations 1–6). Specifically, DO was first enriched at the electrode surface by CTAB@cMWCNTs and is then electrochemically reduced to O_2_^•−^ under cathodic polarization (Equation 1). Meanwhile, luminol (LH_2_) losed one proton to form luminol anion LH^−^ (Equation 2), which was also accumulated at the electrode interface and oxidized to L^•−^ under anodic polarization (Equation 3). Subsequently, O_2_^•−^ rapidly reacted with L^•−^ to form the highly unstable intermediate LO_2_^2−^ (Equation 4). This intermediate then decomposed rapidly to generate excited-state 3-aminophthalate (AP^2−^) with N_2_ release (Equation 5). Finally, AP^2−^ returned to its ground state, accompanied by emission (Equation 6) (Lu et al., 2026; Zhao et al., 2025; Zhou et al., 2025).

$$O_{2}+e^{‐}\;\to \;O_{2}^{•-}$$ (1)

$${\text{LH}}_{2}-H^{+}\;\to \;{\text{LH}}^{-}$$ (2)

$${\text{LH}}^{-}-2e^{-}\;\to \;L^{•-}+H^{+}$$ (3)

$$L^{•-}+O_{2}^{•-}\;\to \;{\text{LO}}_{2}^{2-}$$ (4)

$${\text{LO}}_{2}^{2-}\;\to \;{\text{AP}}^{2-*}+N_{2}$$ (5)

$${\text{AP}}^{2-*}\;\to \;{\text{AP}}^{2-}+h\nu$$ (6)

### 3.5 Enhanced ECL signal and optimization of sensor fabrication and ECL testing

**FIGURE 4:**
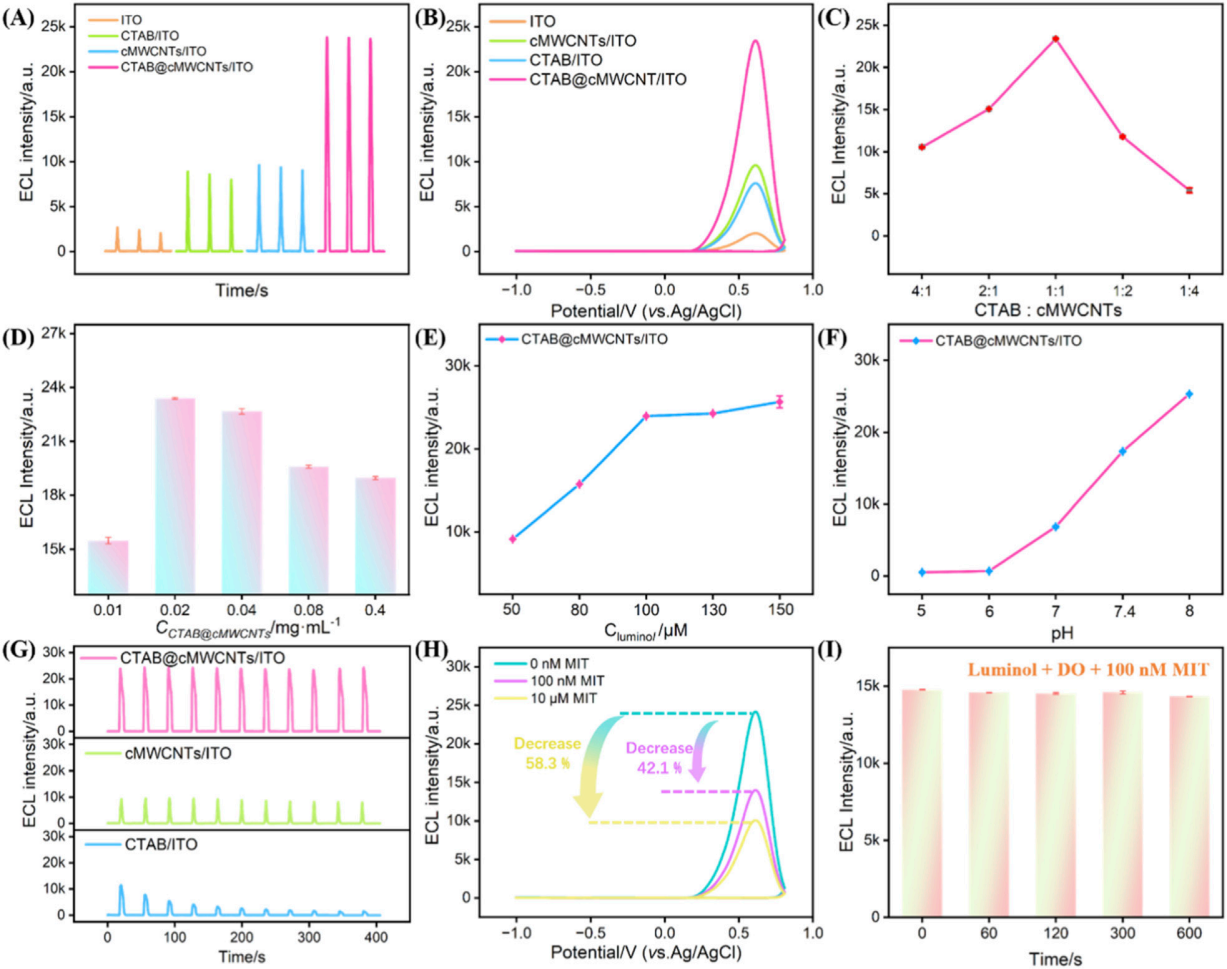
**(A)** ECL intensities and **(B)** intensity-potential profiles of different electrodes in PBS (0.01 M, pH 7.4) + 100 μM luminol. ECL intensities of CTAB@cMWCNTs/ITO electrodes prepared with **(C)** different CTAB/cMWCNTs ratios and **(D)** different CTAB@cMWCNTs concentrations in 0.01 M PBS (pH 7.4) + 100 μM luminol. **(E)** ECL intensities obtained in PBS containing different concentrations of luminol. **(F)** ECL intensities at different pH values. PMT voltage of 600 V to avoid high signal at high pH. **(G)** Stability comparison of three different electrodes. **(H)** ECL intensities of the CTAB@cMWCNTs/ITO electrode after adding different concentrations of MIT into the testing solution. **(I)** ECL responses of the CTAB@cMWCNTs/ITO electrode after enrichment in MIT-containing PBS for different times. PMT voltage: 650 V.

Figures 4A,B compared the ECL responses of different electrodes in PBS (0.01 M, pH 7.4) containing luminol (100 μM), together with the corresponding ECL intensity-potential profiles. The CTAB@cMWCNTs/ITO electrode exhibited the strongest ECL emission, which was remarkably higher than those obtained at ITO electrode, CTAB/ITO electrode, and cMWCNTs/ITO electrode. This resulted from the enrichment of the ECL emitter and co-reactant and facilitated interfacial electron transfer by cMWCNTs.

To achieve optimal analytical performance, the mass ratio of CTAB to cMWCNTs, the concentration of the CTAB@cMWCNTs dispersion, the luminol concentration in the testing solution, and the solution pH were optimized. With increasing cMWCNTs content, the ECL intensity initially increased and then decreased (Figure 4C). This phenomenon can be attributed to the enhanced interfacial electron transfer induced by an appropriate amount of cMWCNTs, thereby improving the ECL response. However, when the proportion of cMWCNTs was excessively high, the dispersion of cMWCNTs became poorer, and the relatively low CTAB content weakened the enrichment capability of the CTAB@cMWCNTs composite, resulting in a decreased ECL signal. Thus, a CTAB/cMWCNTs mass ratio of 1:1 was selected. The concentration of the CTAB@cMWCNTs dispersion was further optimized (Figure 4D). When the composite concentration was lower than 0.02 mg mL^-1^, ECL signal gradually increased with increasing concentration, indicating that, within this low-concentration range, increasing the amount of composite enhanced the local enrichment of luminol and DO at electrode. However, when the composite concentration exceeded 0.02 mg mL^-1^, the ECL signal gradually decreased, probably because thick modification layer might absorb part of the emitted light. Accordingly, 0.02 mg mL^-1^ CTAB@cMWCNTs dispersion was used.

The effects of luminol concentration and solution pH on ECL response were also investigated (Figures 4E,F). The ECL signal of CTAB@cMWCNTs/ITO electrode tended to plateau when luminol concentration reached 100 μM, indicating that the enrichment of luminol and DO reached equilibrium. Thus, 100 μM luminol was selected. In addition, the ECL intensity increased significantly with increasing pH, which can be attributed to the fact that alkaline conditions generally facilitate luminol ionization and DO electrochemistry. Considering that the electrode already exhibited strong ECL response at pH 7.4 and that excessively high pH values might affect the stability of MIT in solution, physiological pH 7.4 was chosen for MIT detection.

### 3.6 Interfacial stability of the sensor and feasibility for MIT detection

Electrode stability is critical for practical sensing applications. As shown in Figure 4G, under continuous cyclic potential scanning, ECL signal of CTAB/ITO electrode gradually decreased. This was because CTAB modified on the electrode surface can enrich DO and luminol from the testing solution, thereby increasing their interfacial concentrations and producing a relatively high ECL signal. However, CTAB has poor stability on the ITO surface and may gradually desorb from the electrode during operation, leading to continuous signal decrease. Although cMWCNTs/ITO electrode exhibited a relatively stable signal, its overall ECL intensity was low. In comparison, the CTAB@cMWCNTs/ITO electrode maintained a strong and highly stable ECL response over 11 consecutive cycles, effectively combining the enrichment capability of CTAB with the stability and excellent conductivity of cMWCNTs. Compared with the single-component CTAB/ITO and cMWCNTs/ITO electrodes, the CTAB@cMWCNTs/ITO composite electrode not only generated a high ECL signal but also significantly improved stability during continuous scanning.

MIT is a synthetic anthraquinone compound and a first-line antitumor drug commonly used in clinical treatment. In the luminol-DO ECL system, phenolic hydroxyl groups in MIT can effectively consume ROS such as O_2_^•−^ generated in the system, suppressing the reaction between O_2_^•−^ and L^•−^ and consequently decreasing the ECL intensity. Thus, MIT detection can be achieved through ECL quenching. As shown in Figure 4H, after adding 100 nM MIT to the testing system, the ECL intensity significantly decreased. When the MIT concentration was further increased to 10 μM, the ECL signal further decreased. These results demonstrate that MIT exhibits an obvious quenching effect on this ECL system, and the quenching efficiency increases with increasing MIT concentration.

To evaluate the response speed of the sensor for MIT detection, the ECL and electrochemical responses of the electrode were examined after immersion in MIT-containing testing solutions for different times. After adding 100 nM MIT to the luminol-DO ECL system, ECL signal of CTAB@cMWCNTs/ITO electrode showed no obvious dependence on enrichment time (Figure 4I). These results indicated that the constructed CTAB@cMWCNTs/ITO electrode enabled rapid MIT detection. The composite electrode can generate stable signal response toward MIT without long-term pre-enrichment, demonstrating advantage for rapid analysis.

### 3.7 Analytical performance of the CTAB@cMWCNTs/ITO electrode for ECL detection of MIT

**FIGURE 5:**
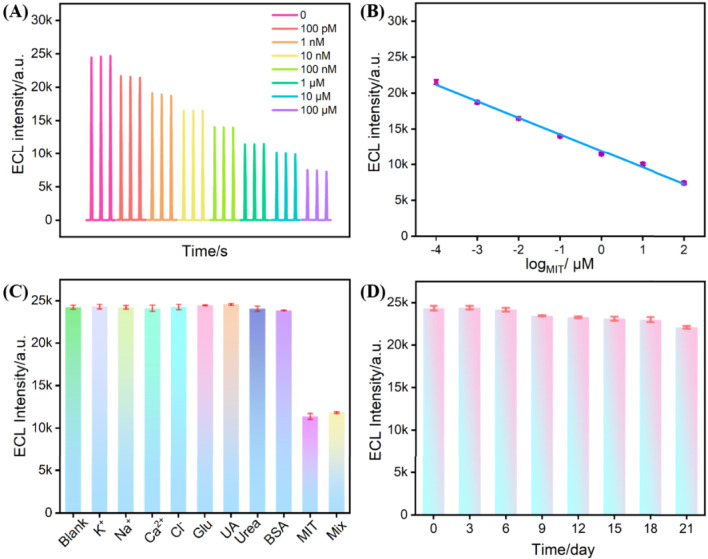
**(A)** ECL responses of CTAB@cMWCNTs/ITO electrode with different concentrations of MIT. **(B)** The corresponding calibration plot. The error bar represented the standard deviation of three measurements. **(C)** Anti-interference performance of the sensor toward different substances. ECL signal was tested in PBS (0.01 M, pH 7.4) containing luminol (100 μM) after the addition of 1 mM K^+^, Cl^−^, Na^+^, Ca^2+^, or glucose (Glu), 0.1 mM uric acid (UA), 2 mM urea, 2 mg/mL bovine serum albumin (BSA), 1 μM MIT, and their mixed solution. *I* and *I*_0_ was ECL signals without and with interferents, respectively. **(D)** ECL signals of the sensor after storage at room temperature for different days.

CTAB@cMWCNTs/ITO electrode was used for ECL detection of MIT with different concentrations to evaluate the analytical performance of the proposed sensor. The ECL detection results were shown in Figure 5A. With increasing MIT concentration, the ECL signal decreased. This signal decrease was attributed to the effective consumption of ROS by MIT and consequently resulted in significant ECL quenching. ECL intensity (*I*_ECL_) exhibited a good linear relationship with the logarithm of MIT concentration (log *C*_MIT_) over the range of 100 pM-100 μM (*I*_ECL_ = −2,311 (±66) log *C*_MIT_ (μM) + 11,923 (±148), *R* ^2^ = 0.995, Figure 5B). The limit of detection (LOD) was calculated based on IUPAC definition (*X*_L_ = *X*_B_ + *kS*_B_), where *X*_L_ was the minimum detectable signal, *X*_B_ and *S*_B_ were the mean and standard deviation of the blank measurements, respectively. Using this value in the calibration plot, the LOD was calculated to be 4.8 pM at a signal-to-noise ratio of 3 (S/N = 3, *k = 3*). Supplementary Table S1 compared MIT detection performance using different methods. Our ECL detection using CTAB@cMWCNTs/ITO electrode exhibited wide linear detection range and low LOD. Thus, the fabricated ECL sensor possessed simple preparation, easy operation and high sensitivity, showing potential for MIT detection with high performance.

To evaluate the selectivity of the sensor, the effects of several potential interfering substances on the ECL signal were investigated. Specifically, K^+^, Cl^−^, Na^+^, Ca^2+^, and Glu, UA, urea, BSA were separately added to the testing system. As shown in Figure 5C, no obvious ECL signal changes were observed after the addition of these interfering species. In contrast, the ECL signal decreased significantly upon the addition of 1 μM MIT. These results demonstrated that the constructed sensor possessed good selectivity and anti-interference capability for MIT detection. The storage stability of the sensor was also examined by storing the prepared electrodes at room temperature for 21 days. The ECL signal retained 90.1% of its initial intensity after storage, indicating good storage stability of the electrode (Figure 5D). The reproducibility of the sensor was evaluated. Five parallel-fabricated independently prepared CTAB@cMWCNTs/ITO electrodes were used to perform parallel detection of MIT (1 μM). An intra-batch reproducibility with RSD of 2.7% was observed. The inter-batch reproducibility of the developed sensor was evaluated by five electrodes prepared from different batches. The RSD for MIT (1 μM) detection was 3.9%.

### 3.8 Real sample analysis

The practicability and reliability of the CTAB@cMWCNTs/ITO electrode for MIT detection in real samples was investigated. Standard addition method was employed to determine MIT in human serum samples. MIT in original serum was not detected. As summarized in Table 1, the recoveries of MIT in spiked human serum samples ranged from 98.5% to 107%, with relative standard deviations (RSD) below 5%. These results indicate that the developed sensor has potential for MIT detection in complex serum matrices.

**TABLE 1:** MIT detection in human serum sample using the fabricated sensor.

Sample	Added (μM)	Found (μM)	RSD (%, n = 3)	Recovery (%)
human serum	1.00	1.05	1.8	105
	10.0	9.85	1.9	98.5
	100	107	1.1	107

## 4 Conclusion

In summary, an ECL sensor based on CTAB@cMWCNTs composite-modified ITO electrode was developed for MIT detection. The proposed sensing platform integrates the enrichment capability of CTAB toward luminol and dissolved oxygen with the excellent electrical conductivity of cMWCNTs, as proved by CV, EIS and ECL results. MIT quenches the luminol-DO ECL system by interacting with ROS in the ECL process, leading to ECL signal decrease. The ECL detection exhibited low limit of detection. The sensor also showed good selectivity, fabrication reproducibility, and storage stability. For real-sample analysis, MIT could be reliably detected in serum samples using the standard addition method. Thus, the CTAB@cMWCNTs-based ECL sensing strategy provides a promising approach for sensitive MIT determination in serum matrix.

## Data Availability

The raw data supporting the conclusions of this article will be made available by the authors, without undue reservation.
