# Supplementary material for: Triple-enrichment interface enabled by surfactant-modified carbon nanotube for signal-off electrochemiluminescence detection of antitumor drug mitoxantrone
Source: Front Chem. 2026 Jul 23;14:1897134. doi: 10.3389/fchem.2026.1897134 (PMC13443035; doi:10.3389/fchem.2026.1897134)
Supplement: Supplementary file 1 [file DataSheet1.docx]

**S1. Chemicals and materials.**

Cetyltrimethylammonium bromide (CTAB, 99%), sodium dihydrogen phosphate dihydrate (NaH_2_PO_4_·2H_2_O, 99%), disodium hydrogen phosphate dodecahydrate (Na_2_HPO_4_·12H_2_O, 99%), sodium bicarbonate (NaHCO_3_, 99.8%), sodium hydroxide (NaOH, 98%), luminol (98%), tert-butanol (TBA, 99%), p-benzoquinone (BQ, 99%), mitoxantrone (MIT, 99%), glucose (Glu, 98%), uric acid (UA, 99%), urea (Urea, 99%), bovine serum albumin (BSA, 98%), and carboxylated multi-walled carbon nanotubes (cMWCNTs, 95%) were purchased from Aladdin Biochemical Technology Co., Ltd (Shanghai, China). Acetone (99.5%), hydrogen peroxide (H_2_O_2_, 30%), absolute ethanol (99.8%), and concentrated hydrochloric acid (HCl, 38%) were obtained from Shuanglin Chemical Reagent Co., Ltd (Hangzhou, China). Sodium chloride (NaCl, 99.99%), sodium nitrate (NaNO_3_, 99.99%), potassium chloride (KCl, 99%), and calcium chloride (CaCl_2_, 94%) were purchased from Gaojing Fine Chemical Co., Ltd (Hangzhou, China). The phosphate buffer solution (PBS, 0.01 M, pH 7.4) was prepared using Na_2_HPO_4_ and NaH_2_PO_4_. All chemicals and reagents used in this experiment were of analytical grade (AR) and used without further purification. All aqueous solutions were prepared with ultrapure water (18.2 MΩ·cm). ITO-coated glass electrodes were purchased from Zhuhai Kaivo Optoelectronic Technology Co., Ltd (Zhuhai, China).

**S2. Measurements and instrumentations.**

X-ray photoelectron spectroscopy (XPS, Thermo Scientific K-Alpha, USA) was employed to characterize the chemical compositions of cMWCNTs, CTAB, and CTAB@cMWCNTs. The morphologies of cMWCNTs and CTAB@cMWCNTs were characterized using transmission electron microscopy (TEM, HT7700, Hitachi, Japan). Scanning electron microscopy (SEM) images were obtained using a Sigma500 scanning electron microscope (Zeiss, Germany). TEM samples were prepared by a dip-coating method using copper grids immersed in the dispersions containing cMWCNTs or CTAB@cMWCNTs. Electrochemical experiments, including cyclic voltammetry (CV), were carried out on an Autolab electrochemical workstation (PGSTAT302N, Metrohm, Switzerland). All electrochemical and ECL measurements were performed using a conventional three-electrode system, consisting of an Ag/AgCl electrode as the reference electrode, a platinum wire or platinum sheet as the counter electrode, and a bare or modified ITO electrode as the working electrode. The scan rate for CV measurements was set at 100 mV/s. Unless otherwise specified, the photomultiplier tube (PMT) voltage for all ECL measurements was maintained at 650 V.

**S3 Supplementary table**

Table S1. MIT detection performance obtained using different methods.

| Materials | Analytical Methods | Linear range | LOD | Ref |
| --- | --- | --- | --- | --- |
| carbon dots | FL | 0.096–30 µM | 0.032 µM | [1] |
| PTh-MWCNT | DPV | 1–100 μM | 8.92 nM | [2] |
| MIP@rQDs@SiO_2_ | Ratiometric FL | 0.2 M–2.0 M | 67 µM | [3] |
| TCPP-His@ZIF-8 MOF | FL | 0.125–14.0 μM | 91.93 nM | [4] |
| Bi film plated GCE | DPV | 0.27–1.0 µM | 0.081 µM | [5] |
| CTAB@cMWCNTs/ITO | ECL | 100 pM–100 μM | 4.8 pM | This work |

PTh-MWCNT: polythiophene polymer-multiwalled carbon nanotubes composite; FL: fluorescence; DPV: differential pulse voltammetry; MIP@rQDs@SiO_2_: molecularly imprinted polymer coated quantum dots; TCPP-His@ZIF-8: tetrakis (4-carboxyphenyl) porphyrin grafted on L-histidine modified ZIF-8 metal-organic framework; GCE: glassy carbon electrode;

GO: graphene Oxide; GCE: DPV: Differential Pulse Voltammetry; CD: carbon dots.

**S4 Supplementary references**

[1] Zhong, Y., Chen, A., Yin, X., Li, R., Deng, Q., Yang, R. (2023). Red emission carbon dots for mitoxantrone detection. *Sens. Actuators B: Chem.* 382, 133535.

[2] Miya, L., Gill, A.A.S., Adu, D.K., Alake, J., Ike, B.W., Govender, P., et al. (2025). Simple PTh-MWCNT nanocomposite electrochemical sensor for detection and quantification of mitoxantrone. *ChemistrySelect* 10, e00830.

[3] Wei, J.-R., Chen, H.-Y., Zhang, W., Pan, J.-X., Dang, F.-Q., Zhang, Z.-Q., et al. (2017). Ratiometric fluorescence for sensitive and selective detection of mitoxantrone using a MIP@rQDs@SiO_2_ fluorescence probe. *Sens. Actuators B: Chem.* 244, 31-37.

[4] Wu, P., Jiao, Y., Cheng, L., Yao, C., Chen, X. (2026). Development of a near-infrared fluorescence sensing platform based on TCPP-His@ZIF-8 for highly sensitive mitoxantrone detection. *Spectrochim. Acta A* 354, 127619.

[5] El-Shahawi, M.S., Khraibah, N.H., Abduljabbar, T.N. (2024). Exploring the potential of plating solution on the fabrication of ex-situ Bi film plated glassy carbon electrode for detection of mitoxantrone drug residue. *Microchem. J.* 205, 111146.
